# Structure of human RNA *N*^6^-methyladenine demethylase ALKBH5 provides insights into its mechanisms of nucleic acid recognition and demethylation

**DOI:** 10.1093/nar/gku085

**Published:** 2014-01-30

**Authors:** WeiShen Aik, John S. Scotti, Hwanho Choi, Lingzhi Gong, Marina Demetriades, Christopher J. Schofield, Michael A. McDonough

**Affiliations:** Department of Chemistry, Chemistry Research Laboratory, University of Oxford, 12 Mansfield Road, Oxford, OX1 3TA, UK

## Abstract

ALKBH5 is a 2-oxoglutarate (2OG) and ferrous iron-dependent nucleic acid oxygenase (NAOX) that catalyzes the demethylation of *N*^6^-methyladenine in RNA. ALKBH5 is upregulated under hypoxia and plays a role in spermatogenesis. We describe a crystal structure of human ALKBH5 (residues 66–292) to 2.0 Å resolution. ALKBH5_66–292_ has a double-stranded β-helix core fold as observed in other 2OG and iron-dependent oxygenase family members. The active site metal is octahedrally coordinated by an HXD…H motif (comprising residues His204, Asp206 and His266) and three water molecules. ALKBH5 shares a nucleotide recognition lid and conserved active site residues with other NAOXs. A large loop (βIV–V) in ALKBH5 occupies a similar region as the L1 loop of the fat mass and obesity-associated protein that is proposed to confer single-stranded RNA selectivity. Unexpectedly, a small molecule inhibitor, IOX3, was observed covalently attached to the side chain of Cys200 located outside of the active site. Modelling substrate into the active site based on other NAOX–nucleic acid complexes reveals conserved residues important for recognition and demethylation mechanisms. The structural insights will aid in the development of inhibitors selective for NAOXs, for use as functional probes and for therapeutic benefit.

## INTRODUCTION

Nucleic acid modifications are found in all forms of life and play key roles in the regulation of gene expression (1). More than 100 different types of post-transcriptional modifications have been identified in RNA (2–4); many with unassigned functions. Some RNA modifications have been intensively studied, e.g. 5′ RNA 7-methylguanosine cap [m^7^G(5′)ppp(5′)N] in messenger RNA (mRNA) (5), but nonetheless, emerging data support the proposal that modifications to RNA constitute an important general mechanism for the regulation of gene expression in both healthy and diseased cells (4,6).

The most abundant internal modification observed in eukaryotic mRNA is methylation of adenine to give *N*^6^-methyladenine (m^6^A) (7). Recent advances in high-throughput sequencing methods have enabled more detailed analysis of this modification in cells (8,9). The context-dependent physiological roles of m^6^A are currently being explored, and small molecule tools to probe the functions of this modification will be useful. The *S*-adenosyl methionine-dependent methyltransferase-like 3, METTL3/MT-A70, catalyzes mRNA adenine *N*^6^-methylation (10). Potential m^6^A binding proteins (‘readers’) have been identified, including (embryonic lethal, abnormal vision, *Drosophila*)-like 1 (ELAVL1), YTH domain family member 2 (YTHDF2) and YTH domain family member 3 (YTHDF3) (8). Two human 2-oxoglutarate (2OG) and iron-dependent oxygenase enzymes, the fat mass and obesity-associated protein (FTO) and AlkB homologue 5 (ALKBH5) have been found to catalyze m^6^A demethylation (11,12), indicating that RNA methylation/demethylation is a dynamic modification.

2OG and iron-dependent oxygenases are widely distributed in aerobic and facultative anaerobic life forms (13–15). 2OG oxygenases use Fe(II) as a co-factor and 2OG and molecular oxygen as co-substrates to catalyze a broad range of chemical reactions including epimerizations, cyclizations, desaturations and hydroxylations (16). In humans, >60 2OG oxygenases have been identified, which have diverse cellular functions including in hypoxia sensing, collagen stabilization, fatty acid metabolism, RNA splicing and epigenetics (17). 2OG oxygenases are structurally characterized by having a core double-stranded β-helix-fold (DSBH), which acts as a scaffold for a conserved HXD/E…H triad of residues. Together with water molecules and/or cosubstrates, the side chains of these residues octahedrally coordinate the Fe(II) cofactor. The 2OG cosubstrate binds between the major and minor β-sheets of the DSBH and occupies two of the six active site metal coordination sites (14). 2OG oxygenase substrates are recognized by various structural elements within and surrounding the active site (13).

A subfamily of 2OG oxygenases acts on nucleic acids (nucleic acid oxygenases [NAOXs]). AlkB was the first 2OG oxygenase to be characterized as an *N*-methylated nucleic acid demethylase (18,19). In *Escherichia coli* (and other bacteria), AlkB is induced on exposure to toxic alkylating agents such as methyl methanesulfonate and enables DNA repair by catalyzing demethylation of 1-methyladenine (m^1^A) and 3-methylcytosine (m^3^C) lesions (18–20). Human homologues of AlkB have been identified: AlkB homologues 1–8 (ALKBH1-8) and FTO (21–24). ALKBH1 was demonstrated to have abasic or apurinic/apyrimidinic (AP) lyase activity independent of 2OG and Fe(II), although no nucleic acid demethylase activity for it has yet to be reported (25,26). ALKBH2 demethylates m^1^A and m^3^C of double-stranded DNA (dsDNA), while ALKBH3 selectively catalyzes demethylation of m^1^A and m^3^C in single-stranded DNA (ssDNA) (23,24,27,28). ALKBH8 catalyzes hydroxylation of 5-methoxycarbonylmethyluridine (5mcmU) on transfer RNA (tRNA), and the JmjC domain oxygenase TYW5 hydroxylates tRNA wybutosine (yW) (29–32). Both 5mcmU and yW are modifications to bases at the wobble position of tRNA. Other 2OG oxygenases acting on nucleic acid substrates have been identified, including the ten-eleven translocation enzymes (TETs 1–3), which oxidize 5-methylcytosine (5mC) to sequentially form 5-hydroxymethylcytosine (5hmC), 5-formylcytosine (5fC), and 5-carboxycytosine (5caC) (33–35).

Following from pioneering structural work on NAOXs preferentially acting on *N*-methylated DNA, i.e. AlkB and subsequently ALKBH2/3 (27,28,36,37,38), structures of NAOXs acting on RNA including those of ALKBH8, FTO and TYW5 have been reported (32,39,40). However, to date, the structures of FTO (39,41) are the only ones reported for a NAOX that preferentially catalyzes *N*-demethylation of methylated mRNA. FTO is a potential target for obesity (42,43), and various groups are working to develop selective inhibitors for FTO (41,44), both as functional probes and for target validation. Hence, structural studies on ALKBH5, a NAOX that, like FTO, catalyzes the demethylation of *N*-methylated mRNA, are of both basic and pharmaceutical interest. Consequently, we undertook structural studies on ALKBH5 with a view to comparing it with FTO.

**Scheme 1.**
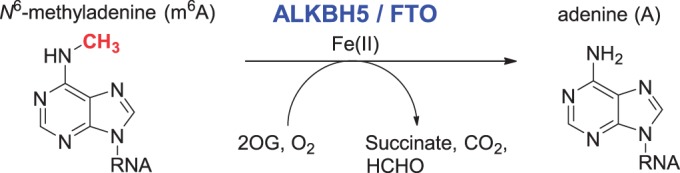
ALKBH5 and FTO catalyze m^6^A demethylation.

It was recently reported that ALKBH5 (Uniprot entry Q6P6C2) is localized to the nucleus, has 2OG and iron-dependent activity and is upregulated under hypoxic conditions by the hypoxia-inducible factor (HIF) transcription factor pathway (45). ALKBH5 was observed to be highly expressed in the lung, followed by testis, pancreas, spleen and ovary (46). It was also reported that ALKBH5 localized to nuclear speckles and that decreased ALKBH5 levels affect mRNA export and processing, leading to altered spermatogenesis (12). High-throughput analyses have provided evidence for multiple post-translational modifications to ALKBH5, including serine- and tyrosine-residue phosphorylation as well as alanine- and lysine-residue acetylation (47–50). Importantly, further work identified m^6^A in single-stranded RNA (ssRNA) as a substrate for ALKBH5 (12) (Scheme 1); m^6^A in mRNA was also shown to be a substrate for FTO (11).

Here we describe crystallographic studies on ALKBH5 and compare its active site characteristics with those of other reported NAOX structures (27,28,32,36,39,40,51). The results reveal both conserved and distinctive features of ALKBH5 and FTO, which will be useful in the development of selective inhibitors.

## MATERIALS AND METHODS

### Protein expression and purification

A plasmid with the vector backbone pNIC28-Bsa4 encoding a hexahistidine-tagged ALKBH5_66__–__292_ construct was transformed into *E. coli* BL21 (DE3) cells (45). The transformed cells were grown at 37 °C until an OD_600_ of 0.6–0.8 was reached. ALKBH5 expression was then induced with 0.5 mM isopropyl β-D-1-thiogalactopyranoside (IPTG). Cell growth was then continued for 20 h at 18°C. The cells were then harvested by centrifugation (Beckman Avanti J-25, rotor JA10, 7000×*g*, 8 min), and the resultant cell pellets were stored at −80 °C. The frozen cell pellets were thawed and resuspended in 50 mM tris(hydroxymethyl)aminomethane-hydrochloride (Tris-HCl) pH 7.5, 500 mM sodium chloride (NaCl) and 10 mM imidazole and subsequently lysed by sonication on ice. The lysates were then centrifuged (Beckman Avanti J-25, rotor JA25.5, 43 400×*g*, 20 min), and the supernatant was loaded onto a 5-ml HisTrap HP column (GE Healthcare) and purified using an AKTA FPLC system. The column was washed with 50 mM Tris-HCl pH 7.5, 500 mM NaCl and 40 mM imidazole, and the protein was eluted with 50 mM Tris-HCl pH 7.5, 500 mM NaCl and 375 mM imidazole. Ethylenediaminetetraacetic acid (EDTA) was added to the protein solution to a final EDTA concentration of 100 mM and then incubated on ice for 30 min before being buffer-exchanged into 25 mM Tris-HCl pH 7.5, and 100 mM NaCl. The protein was further purified using a (5 ml) HiTrap Heparin column (GE Healthcare) followed by a (20 ml) MonoQ column; in both cases, the protein was eluted with a gradient from 25 mM Tris-HCl pH 7.5, to 25 mM Tris-HCl pH 7.5 and 500 mM NaCl. The purified protein was then buffer-exchanged into 25 mM Tris-HCl pH 7.5 and concentrated to 11 mg/ml for storage at −80 °C.

### Crystallization

Crystallization trials of ALKBH5_66__–__292_ were carried out in the presence of Mn(II) [as a non-reactive Fe(II) substitute] and various known 2OG oxygenase inhibitors (52). ALKBH5_66__–__292_ (MW 28.7 kDa) was crystallized in sitting drops at 293 K by the vapour diffusion method in the presence of Mn(II) and (1-chloro-4-hydroxyisoquinoline-3-carbonyl)glycine (IOX3). Crystallization drops contained 0.2 μl of a protein solution containing a final concentration of 10 mg/ml hexahistidine-tagged ALKBH5_66__–__292_, 0.5 mM manganese(II) chloride and 2 mM IOX3 (41,53) mixed with 0.1 μl of well solution containing 125 mM potassium nitrate and 15% (w/v) polyethylene glycol 3350. Crystals (size ∼100 × 50 × 50 μm) appeared after 3 months. Crystals were harvested using nylon loops and cryoprotected using well solution diluted with 25% (v/v) glycerol and flash-cooled in liquid nitrogen.

### Data collection and structure determination

Data (0.2° oscillation/image, 180° total rotation) were collected at 100 K on a single crystal at Diamond synchrotron beamline I24 using a wavelength of 0.97889 Å and a Pilatus3 6 M detector. All data were indexed, integrated and scaled using HKL3000 (54). The PHENIX (55) subroutine ENSEMBLER (56) was used to generate an ensemble using the structures of human homologues of AlkB: FTO (PDB ID 4IE5), ALKBH2 (PDB ID 3S57), ALKBH3 (PDB ID 2IUW) and ALKBH8 (PDB ID 3THT) to be used as a search model for phasing by molecular replacement. The structure was then solved by molecular replacement using PHASER (56). An initial model of ALKBH5 was generated by PHENIX AUTOBUILD (57), which included 411 residues (∼97% of the final number of observed residues making up the two molecules in the asymmetric unit). Iterative cycles of model building and refinement were performed using COOT (58) and PHENIX (55) until converging R and R_free_ no longer decreased. See Table 1 for detailed data collection and refinement statistics.

**Table 1.** Crystallographic data collection and refinement statistics

Structure	ALKBH5_66–292_
PDB ID	4NJ4
Radiation source	Diamond I24
Resolution range (Å)	50.00–2.02 (2.09–2.02)^a^
Space group	*P* 2 2_1_ 2_1_
Unit cell dimensions	
* a*, *b*, *c* (Å)	67.1, 82.7, 89.2
α, β, γ (°)	90, 90, 90
Total number of reflections observed	199 163
Number of unique reflections	33 274
Redundancy	6.0 (5.6)^a^
Completeness (%)	100.0 (99.9)^a^
*I/σ(I)*	5.9 (2.0)^a^
*R*_merge_^b^	0.142
Wilson B factor (Å^2^)	30.22
R_work_ (%)^c^	16.46
*R*_free_ (%)^d^	21.81
RMS deviation from ideality	
Bonds	0.010 Å
Angles	1.190°
Average B factor (Å^2^)	40.53
Number of water molecules	224

^a^Outermost shell.

^b^R_merge_ = ∑_j_∑_h_| *I*_hj_ − <*I*_h_>|/∑_j_∑_h_ <*I*_h_>.

^c^R_work_ = ∑||Fobs| − |Fcalc||/|Fobs| × 100.

^d^R_free_, based on 10% of the total reflections.

### Activity assays

*In vitro* demethylation assays (12) were performed in triplicate in a 50 μl reaction mixture containing 4 μM ALKBH5_66__–__292_, varied concentrations of 5-mer ssRNA (10, 20, 50, 100 and 200 μM) with the sequence 5′-GGm^6^ACU-3′ (ELLA Biotech, Munich, Germany), 300 μM 2OG, 2 mM l-ascorbate, 150 μM diammonium Fe(II) sulfate complex and 25 mM Tris-HCl, pH 7.5. The reaction mixtures were incubated at room temperature, and 2 μl of sample from each reaction was quenched with 2 μl of 20% (v/v) formic acid at specific time points. One microlitre of each quenched sample was then mixed with 1 μl of matrix-assisted laser desorption ionization (MALDI) matrix made up of two parts 0.5 M 2,4,6-trihydroxyacetophenone in ethanol and one part 0.1 M ammonium citrate dibasic in water. The relative quantities of product and substrate were analysed using MALDI-ToF mass spectrometry (MS) (Supplementary Figure S1a). The Michaelis–Menten curve was fit using non-linear regression, and the K_m_ of the substrate was estimated using GraphPad Prism (Supplementary Figure S1b).

Inhibition assays were performed in triplicate for each inhibitor in a 25 μl reaction mixture (final volume) containing 4 μM ALKBH5_66-292_, 80 μM 5-mer ssRNA with the sequence 5′-GGm^6^ACU-3′, 150 μM 2OG, 2 mM l-ascorbate, 150 μM diammonium Fe(II) sulphate complex, 150 μM inhibitor [*N*-oxalylglycine (NOG), 2,4-pyridinedicarboxylate (2,4-PDCA) or IOX3] and 25 mM Tris-HCl, pH 7.5. Controls in triplicate without inhibitors were also set up. Reactions were incubated at room temperature and quenched after 5 min with an equivolume of 20% (v/v) formic acid and analyzed using MALDI-ToF MS.

### ALKBH5 Cys200-IOX3 MS

A 0.2 μl crystallization drop mixture incubated for 16 weeks containing an ALKBH5 crystal was first dissolved in 1 μl of 6 M urea in 100 mM Tris-HCl, pH 7.8, and further diluted with 30 μl of the same buffer. Then 1.5 μl of 200 mM dithiothreitol in 100 mM Tris-HCl pH 7.8, was added, and the mixture was vortexed and incubated for 10 min at room temperature. Six microlitres of 200 mM iodoacetamide in 100 mM Tris-HCl pH 7.8, was added, and the mixture was vortexed and incubated for 10 min at room temperature. A further 6 μl of 200 mM dithiothreitol in 100 mM Tris-HCl pH 7.8, was added, and the mixture was vortexed and incubated for an additional 10 min at room temperature. The mixture was then diluted with 230 μl of MilliQ-H_2_O and vortexed. Five microlitres of Glu-C (2 μg/μl in 100 mM Na/K phosphate pH 7.2; Promega, *Staphylococcus aureus* V8, MS grade) was then added to the sample and incubated at 37°C overnight according to the standard procedure (59).

The digested peptides were then purified by first equilibrating a C_18_ Sep-Pak cartridge (Waters, WAT020515) with 5 ml of elution buffer [65% (v/v) acetonitrile and 0.1% (v/v) formic acid in MilliQ-H_2_O], followed by 10 ml of wash buffer [2% (v/v) acetonitrile and 0.1% (v/v) formic acid in MilliQ-H_2_O]. The sample was then loaded onto the column and washed with 10 ml of wash buffer. The column was eluted with 1.5 ml of elution buffer and collected in a 1.5 ml tube. Peptides were dried in a SpeedVac and resuspended in 20 μl of wash buffer for analysis.

Peptides were analyzed using a nanoACQUITY UPLC coupled to SYNAPT HDMS interfaced with a nano-electrospray source (Waters Corporation, Milford, MA, USA). Peptide digests were injected on a 5 μm symmetry C_18_ column (180 μm × 20 mm) and washed for 1 min at 15 μl min^−^^1^ with 0.1% (v/v) formic acid. Peptides were then separated and eluted for MS analysis using a gradient of acetonitrile containing 0.1% (v/v) formic acid at 300 nl min^−^^1^ over 23 min on a nanoACQUITY UPLC column (BEH130 C_18_ 1.7 μm particle size (75 μm inner diameter × 250 mm length). The column temperature was set at 35 °C. The reference for the nanolockspray was set to the doubly charged peak of Glu-Fiprinopeptide B at a concentration of 500 fmol ul-1 flowing at 400 μl min^−^^1^. The reference was constantly infused and sampled at 30 s intervals.

The eluted peptides were analyzed in the positive ionization mode over a mass range of 50–1990 *m/z* with a scan time of 0.6 s. The online-eluted peptides were analysed using an MS^E^ method collecting MS/MS data using collision energy ramping from 15 to 35 eV. Spectra were processed using BioLynx (Waters Corporation, Milford, MA, USA).

### Theoretical modelling of substrate binding

The ALKBH2-dsDNA structure (PDB ID 3BUC) and the AlkB-tri-nucleotide structure (PDB ID 3I2O) were used as templates for modelling binding of a 5-mer ssRNA (sequence: 5′-GGm^6^ACU-3′) into the ALKBH5 active site. The ALKBH2-dsDNA structure was first superimposed on ALKBH5. The protonation states for aspartate, glutamate, histidine and lysine residues were then assigned followed by geometric optimization of the positions of the hydrogen atoms by restrained energy minimization using the OPLS-2005 force-field (60). A GB/SA effective water model (61) was chosen as the solvent model, and non-bonded electrostatic interactions were truncated with a cut-off distance of 20 Å. The convergence process was terminated after 1000 cycles with a 0.05 kcal/mol/Å gradient threshold. After energy minimization, QM/MM calculations (62) were performed to obtain the optimized geometry for the active site metal, His204, His266, Asp206, 2OG and m^6^A-modified ssRNA at the DFT-M06-2X/6-31G** level (63) using a function consisting of a meta-hybrid of the GGA DFT function (64) and the M06 family function adapted for organometallic structures (65).

## RESULTS AND DISCUSSION

ALKBH5_66__–__292_ has been reported as being catalytically active for uncoupled 2OG turnover (45). Following from the report of a longer construct of ALKBH5 (residues 66–394) catalyzing demethylation of m^6^A in a 15-mer ssRNA containing a 5′-GGm^6^ACU-3′ motif (12), we confirmed that our shorter ALKBH5_66__–__292_ construct was also catalytically active and determined kinetic constants using a 5-mer ssRNA substrate with the sequence 5′-GGm^6^ACU-3′ (Supplementary Figure S1a and b). We then carried out end point inhibition assays to investigate whether the enzymatic activity of ALKBH5 can be inhibited by the generic 2OG oxygenase inhibitors NOG, 2,4-PDCA (66) and the more specific compound IOX3, which was originally developed as a prolyl hydroxylase (PHD) inhibitor (52). Relative to no inhibitor controls, all three inhibitors inhibited the activity of ALKBH5_66__–__292_. 2,4-PDCA was the most potent of the three inhibitors tested (9% residual activity), followed by IOX3 (40% residual activity) and NOG (44% residual activity) (Supplementary Figure S2). In contrast, IOX3 was shown to be a more potent inhibitor of FTO than both NOG and 2,4-PDCA (44). IOX3 has been in clinical trials as a HIF PHD inhibitor (52).

### Structure determination of ALKBH5

Following crystallization trials with various 2OG oxygenase inhibitors, we obtained crystals of ALKBH5 in the presence of Mn(II) and IOX3. We then determined a crystal structure of ALKBH5_66__–__292_. The space group was determined to be *P* 2 2_1_ 2_1_, with unit cell constants a = 67.1 Å, b =82.7 Å, c = 89.2 Å; α = β = γ = 90°. The calculated Matthews coefficient (V_m_ 2.2 Å^3^) with a solvent content of 43% indicated two molecules per asymmetric unit (Chain A and Chain B). The structure of ALKBH5_66__–__292_ was solved by molecular replacement using an ensemble of ALKBH2, ALKBH3, FTO and ALKBH8 (final translation function Z score 8.8). The model output from PHASER was based on FTO. AUTOBUILD successfully generated a model of ALKBH5_66__–__292_ with R and R_free_ values of 0.2386 and 0.2714. The ALKBH5_66__–__292_ model was then improved by iterations of manual fitting and refinement to final R and R_free_ values of 0.1646 and 0.2181, respectively.

### Overall ALKBH5 structure

**Figure 1.**
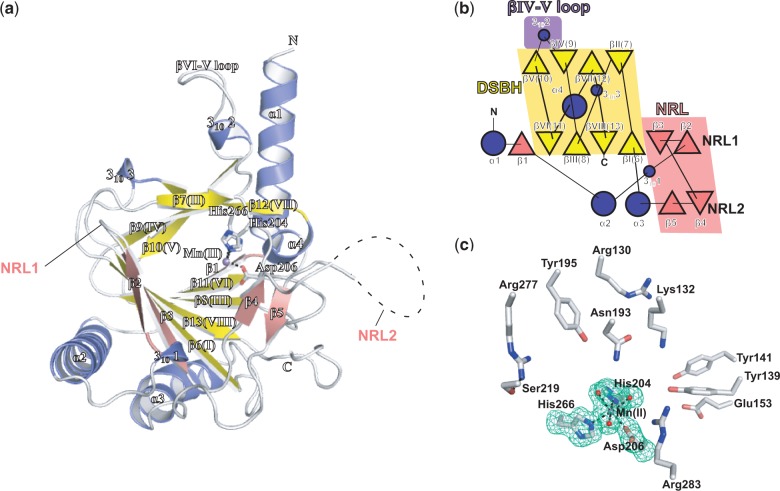

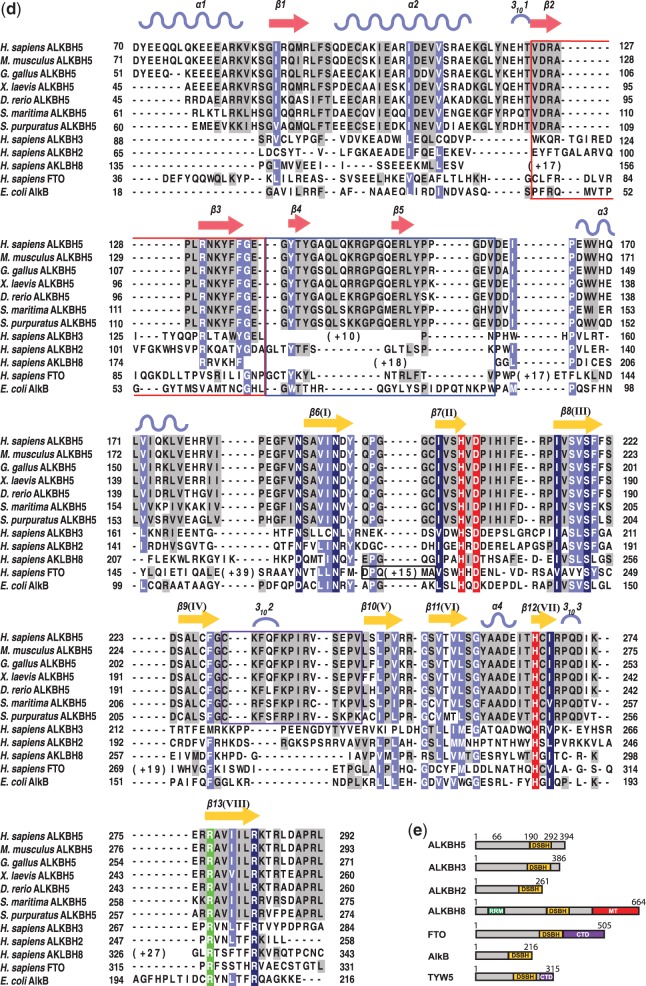
(**a**) Ribbons representation of the ALKBH5_66–292_ structure showing the active site metal Mn(II) (purple sphere) substituting for Fe(II); residues His204, Asp206 and His266 (white sticks); NRL1 and NRL2 (pink fonts); disordered residues (dashed line); the DSBH β-strands I–VIII (yellow); other β-strands (salmon); and α- and 3_10_-helices (blue). (**b**) Topology of the structure of ALKBH5_66–292_. β-strands are shown as triangles, α-helices as large circles and 3_10_-helices as small circles. The βIV-V loop is highlighted in purple, the DSBH in yellow and the NRLs in salmon. (**c**) Active site residues of ALKBH5_66–292_ with representative electron density (3.0σ mF_o_-DF_c_ OMIT; green mesh) for side chains of His204, Asp 206, His266 and water molecules (red spheres) all coordinated (black dashed lines) to the active site Mn(II) ion (purple sphere) (**d**) A ClustalW2 (67) sequence alignment of ALKBH5 homologues from various organisms [from top: *Homo sapiens*: human (sequence identity 100%, PDB ID 4NJ4, Uniprot ID Q6P6C2); *Mus musculus*: mouse (sequence identity 97%, Uniprot ID Q3TSG4); *Gallus gallus*: chicken (sequence identity 86%, F1NIA5); *Xenopus laevis*: frog (sequence identity 78%, Uniprot ID Q6GPB5); *Danio rerio*: zebrafish (sequence identity 72%, Uniprot ID Q08BA6); *Strigamia maritima*: centipede (sequence identity 56%, Uniprot ID T1JJ71); and *Strongylocentrotus purpuratus*: purple sea urchin (sequence identity 52%, Uniprot ID H3I4D7)]; combined with structure-based sequence alignment (68) with ALKBH3 (PDB ID 2IUW); ALKBH2 (PDB ID 3BUC); ALKBH8 (PDB ID 3THT); FTO (PDB ID 3LFM); and AlkB (PDB ID 3I3Q). A few selected manual adjustments were made to the alignment to correct for likely automated errors. *Note:* Variant residues from the reported ALKBH2 (PDB ID 3BUC) structure sequence were changed to reflect the wild-type ALKBH2 sequence (Uniprot Q6NS38). Residues highlighted as conserved (dark blue); semi-conserved (light blue); weakly conserved (grey); conserved 2OG oxygenase catalytic triad HXD…H, red; conserved 2OG binding arginine (green). Boxed residues indicate those forming NRL1 (red); NRL2 (blue); the βIV-V loop of ALKBH5 (purple); the L1 loop of FTO (black). Secondary structural elements of *H. sapiens* ALKBH5 are represented as light blue sinusoidal waves (α-helices), red arrows (β-strands excluding the DSBH core), yellow arrows (β-strands of the DSBH core) and single light blue arcs (3_10_-helix). (**e**) Schematic domain representations of human NAOXs for which structures have been reported. DSBH, double-stranded β-helix core domain; RRM, RNA recognition motif; MT, methyltransferase domain; and CTD, C-terminal domain.

**Figure 2.**
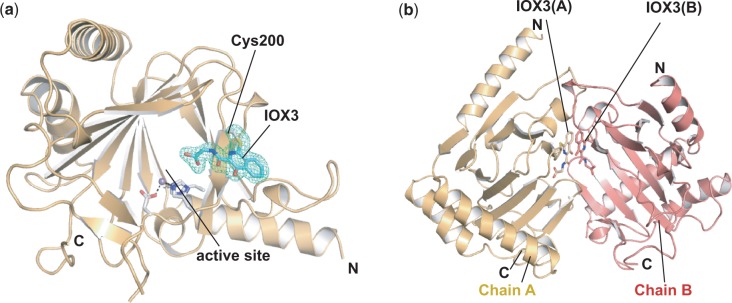
Binding of 1-chloro-4-hydroxyisoquinoline-3-carbonyl)glycine (IOX3) to ALKBH5 involves covalent attachment (Figure S8). (**a**) The small molecule IOX3 (cyan sticks) reacts and forms a covalent bond with the side chain of Cys200 (white sticks); the electron density map (3.0σ mF_o_–DF_c_ OMIT; green mesh) is shown. (**b**) Two protein molecules in an asymmetric unit of an ALKBH5_66–292_ crystal. Covalently attached IOX3 molecules from each protein molecule stack against each other *via* π–π interactions.

As observed in other 2OG oxygenase structures (13,14), the conserved DSBH core fold of ALKBH5 consists of eight anti-parallel β-strands βI–VIII (β6–13), which form two β-sheets: the major β-sheet (strands β6, 8, 11 and 13) and the minor β-sheet (strands β7, 9, 10, 12) (Figure 1a). Three extra β-strands, β1, β2 and β3, extend the major β-sheet. The DSBH is flanked by three helices α1, α2 and α3 (Figure 1b). The DSBH acts as the scaffold for the three Fe(II)-ligating residues His204, Asp206 and His266. Mn(II) is observed bound in the Fe(II) binding site, and the 2OG binding pocket is positioned in a cavity between the two β-sheets of the DSBH with the more open end of the cavity apparently providing substrate access to the active site, a feature common to all 2OG-dependent oxygenases (14). Electron density was observed for 219 residues of Chain A (residues 66–68 and 145–149 were not observed) and 206 residues of Chain B (residues 66–77 and 143–151 were not observed). The structures of Chains A and B are similar with a calculated root-mean-square deviation of 0.31 Å for 206 Cα atoms. The active site openings of both Chains A and B are buried at the interface between the two chains in the asymmetric unit (Figure 2 and Supplementary Figure S3).

**Figure 3.**
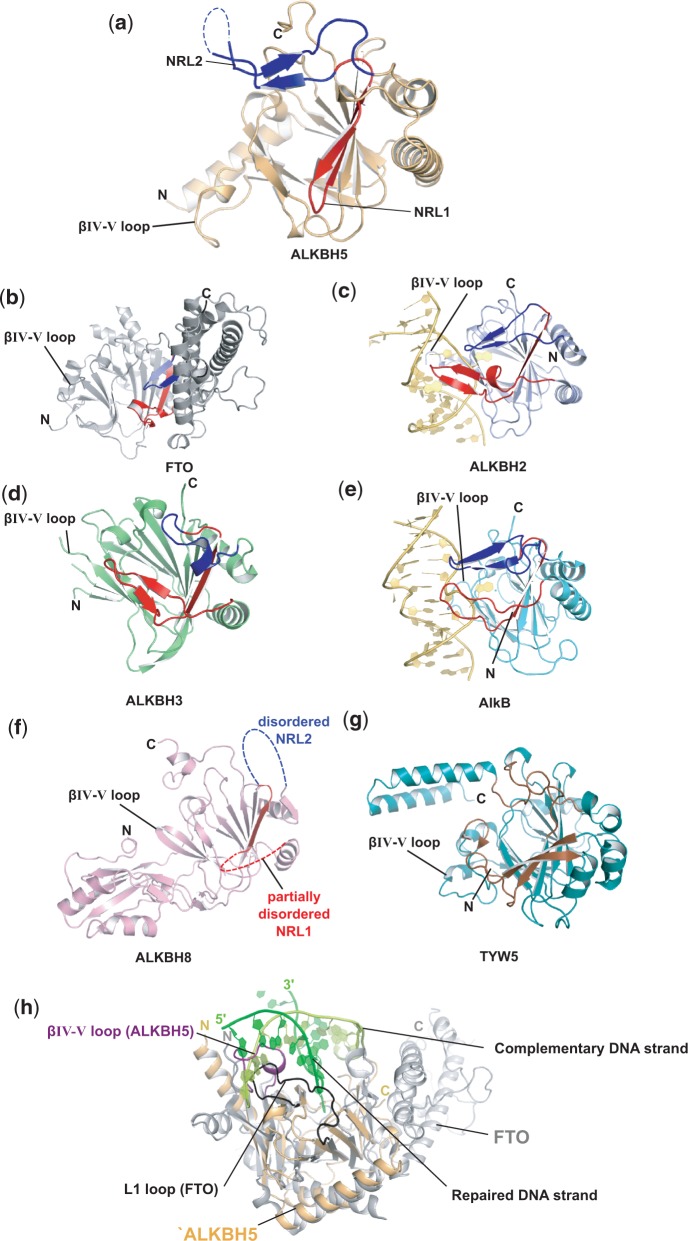
Comparison of NAOX structures reveals differences in their nucleotide recognition lids. NRL1 (red) and NRL2 (blue); (**a**) ALKBH5 (PDB ID 4NJ4), (**b**) FTO (PDB ID 3LFM), (**c**) ALKBH2 in complex with dsDNA (PDB ID 3BUC), (**d**) ALKBH3 (PDB ID 2IUW) and (**e**) AlkB in complex with dsDNA (PDB ID 3BIE). (**f**) The NRL sequences of ALKBH8 (PDB ID 3THT) are mostly disordered. (**g**) TYW5 (PDB ID 3AL5) from the JmjC oxygenase subfamily; note the different structural elements for tRNA substrate recognition; potential substrate contact regions are coloured brown. (**h**) Superimposition of ALKBH5 (light orange ribbon), FTO (grey ribbon) and ALKBH2 (not shown) in complex with double-stranded DNA (green). The βIV-V loop (purple) of ALKBH5 and L1 loop (black) of FTO overlap with the ‘unrepaired’ DNA strand (light green), potentially conferring single-strand selectivity for ALKBH5 and FTO.

The N-terminal region of the ALKBH5_66__–__292_ structure begins with a long α-helix (α1, residues 70–88). Secondary structure prediction (69) for residues 1–66 of ALKBH5 indicates mostly α-helix in this region, which may form a helical bundle with ‘α1’ or possibly a coiled coil motif. A crystal structure of ALKBH8 reveals an extended mixed α/β N-terminal domain, termed an RNA recognition motif, which is believed to be involved in tRNA binding (40). We cannot rule out a similar role for the N-terminal region of ALKBH5_66__–__292_ (partially absent in this construct) in substrate binding. In ALKBH5, the nucleotide recognition lid (NRL), observed in all NAOXs, possesses two β hairpin-like loops β2–3 (NRL1) and β4–5 (NRL2) (see below) (Figure 3a).

A loop between strands βIV and βV (residues 229–242), which contains a 3_10_-helix (3_10_2), extends from the DSBH forming an outer wall of the active site (Figure 1a). The βIV–V loop, which is known to be involved in substrate binding in other 2OG oxygenases (13,70), is apparently constrained by a disulfide bond between Cys230 of the βIV-V loop and Cys267 of DSBH strand βVII (Supplementary Figure S4) and by interactions with the N-terminal helix α1. Interestingly, the side chain of Cys227 is positioned to present a surface-exposed thiol and is adjacent to Cys267; these observations raise the possibility of a thiol/disulfide ‘redox shuffle’ of the Cys230–Cys267 disulfide to form a Cys227–Cys267 disulfide and in turn relax the βIV–V loop conformation (Supplementary Figure S3). The largest difference observed between Chains A and B occurs at the apex of the βIV–V loop between residues 236 and 240. However, this difference may, at least in part, be a consequence of crystal packing due to interactions of this region with neighbouring molecules.

The C-terminal regions of some NAOXs are of functional importance. The tRNA 5-methoxycarbonylmethyluridine hydroxylase ALKBH8 has a Zn(II) bound between its DSBH and the C-terminal region of the oxygenase domain that leads to a C-terminal methyltransferase domain. The helical bundle C-terminal domain of FTO has been shown to be required for FTO activity (39). Our studies show that the C-terminal region of ALKBH5 (residues 293–394) is not essential for m^6^A demethylase activity at least when using 5-mer ssRNA *in vitro* (Supplementary Figure S1a). The C-terminus of ALKBH5 (residues 293–394) is largely predicted to be disordered (69) and contains numerous proline, arginine and serine residues. This is notable because Arg–Ser-rich regions of SR proteins are involved in RNA binding (71). The multiple serine residues (Ser361, Ser371, Ser374, Ser384) flagged as potential phosphorylation sites in ALKBH5 have the potential to regulate RNA interactions.

Modification of Cys200 was observed in electron density maps. The shape of the electron density suggested that it was derived from the ALKBH5 inhibitor, IOX3, used in the crystallization conditions. Subsequent refinement led to assignment of the modification as arising from the reaction of IOX3 with the Cys200 side chain with the loss of chlorine (Figure 2a). Two molecules of the covalently bound IOX3 derivative, each attached to different ALKBH5 molecules at Cys200, are observed to be in proximity (<3.5 Å) with their aromatic bicyclic rings stacked against one another, presumably aiding crystal packing and formation (Figure 2b). Fragmentation MS analysis (MS/MS) of a solubilized ALKBH5 crystal supports the proposal that the modification occurs at Cys200 as observed in the crystallographic analyses (Supplementary Figure S5).

Although structures of IOX3 have been reported in complex with other 2OG oxygenases, including PHD2 and FTO (41,52), its attachment by covalent reaction, as observed with ALKBH5, is unique. In the structural complexes of IOX3 with PHD2 and FTO, IOX3 binds non-covalently to the active site metal in a bidentate manner (41,72,73) (Supplementary Figure S6a). This binding mode is not possible for the Cys200-linked IOX3 in the ALKBH5 structure because it is too far from the active site metal and its position is restrained (Supplementary Figures S6b and S7). It thus seems most likely that the reaction of IOX3 with Cys200 occurred during the prolonged crystallization process (up to 12 weeks) via a nucleophilic aromatic substitution reaction (Supplementary Figure S8). Although the overall results indicate that the crystallographically observed alkylation of Cys200 by IOX3 is likely not relevant to the inhibition of ALKBH5 at the time scale of our inhibition studies in solution (5 min), the observed reaction does raise the question as to whether S-arylation via nucleophilic aromatic substitution may occur during clinical application of C1-chlorinated isoquinoline derivatives. Nucleophilic aromatic substitution of amino acids and residues occurs during derivitization by the Sanger reagent, and was reported for the irreversible inhibition of the thyroid hormone receptor TRβ by methylsulfonylnitrobenzoates (74,75). Detailed inhibition studies of ALKBH5 will be reported in due course.

### Active site

In addition to the conserved HXD…H motif (His204, Asp206, His 266) and arginine (Arg277) from DSBH strand βVIII required for 2OG binding (see below), a combination of hydrophobic and polar residues line the 2OG binding cavity (Ile281, Val279, Tyr195, Ile268, Leu226, Ile201, Asn193 and Val191). The His206Ala ALKBH5 variant was found to be inactive, consistent with an essential role for His206 in catalysis; activity of the His266Ala variant was substantially impaired (12) in agreement with prior studies that show two metal ligating residues can be sufficient for catalytic activity in other 2OG oxygenases (76). The next shell of residues lining the opening to the 2OG binding site includes Arg283, Asp125, Arg130, Tyr141, Phe134, Lys132, Tyr139, Glu153 and Cys200 (Figures 1c and 2a).

**Figure 4.**
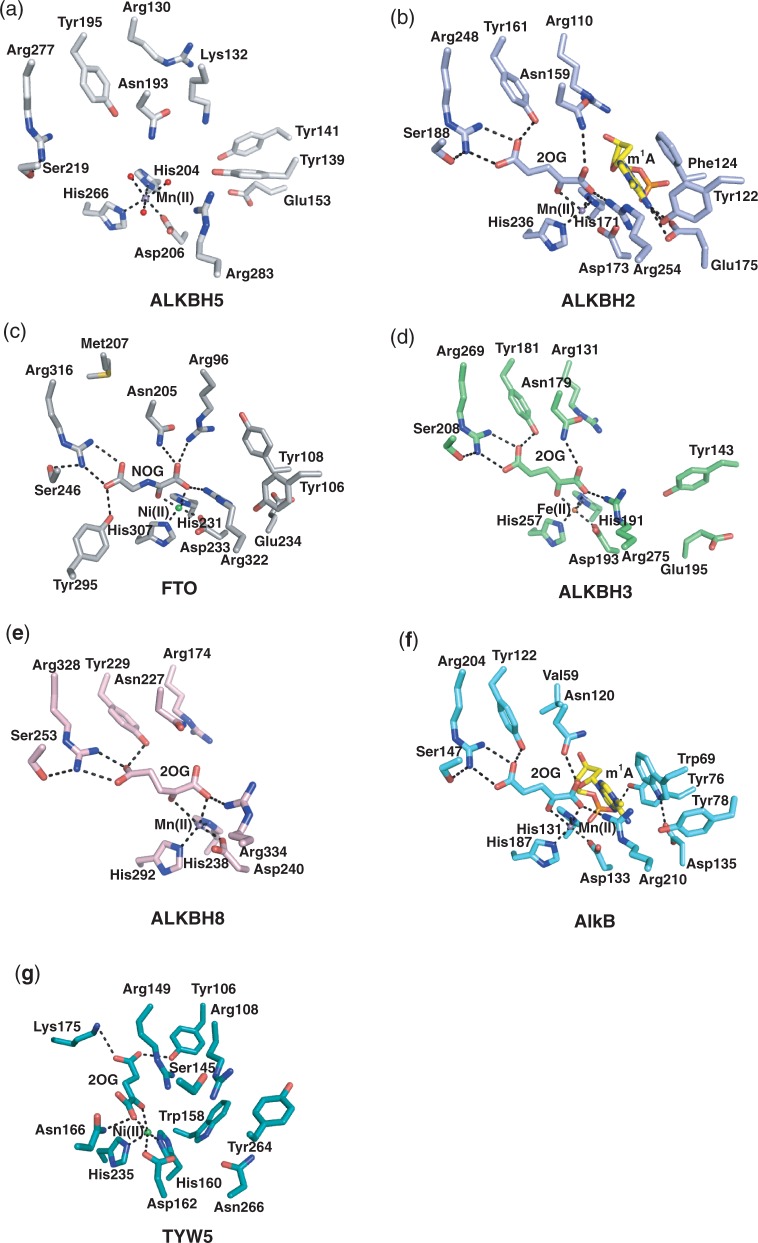
Comparison of the active site of ALKBH5 with those of other nucleic acid oxygenases. Active site residues of (**a**) ALKBH5 (white sticks), (**b**) ALKBH2 (light blue sticks), (**c**) FTO (grey sticks), (**d**) ALKBH3 (green sticks), (**e**) ALKBH8 (pink sticks), (**f**) AlkB (cyan sticks) and (**g**) TYW5 (teal sticks) (PDB ID 3AL6). Oxygen (red), nitrogen (blue), phosphorous (orange), sulphur (yellow), m^1^A base carbon (yellow), Mn(II) (purple sphere), Fe(II) (orange sphere), Ni(II) (green sphere) and water molecule (red sphere) and electrostatic interaction (black dashed line) are indicated.

Studies with other 2OG oxygenases show that differences in the 2OG binding pocket can be exploited for the development of selective inhibitors (52,77). The 2OG binding pocket of ALKBH5 includes residues Arg277 and Tyr195 (Figures 1c and 4a), which likely interact with the C5-carboxylate of 2OG as deduced by comparison of the ALKBH5 structure with those for NAOX–2OG/NOG complexes (27,28,36,39,40) (Figure 4b–f). Interestingly, while FTO possesses the conserved arginine involved in 2OG C5-carboxylate binding as well as a tyrosine residue, the 2OG binding tyrosine (Tyr295) in FTO comes from DSBH βVI (Figure 4c), differentiating it from ALKBH5 and the other AlkB homologues where a 2OG binding tyrosine is derived from DSBH βI.

All of the AlkB-like NAOXs, including ALKBH5, also have a serine residue that interacts with the 2OG C5-carboxylate binding arginine (Figure 4a–f). This serine is not present in the wybutosine tRNA hydroxylase TYW5, the 2OG binding pocket architecture of which is considerably different from those in the AlkB subfamily (27,28,32,36,39,40). Rather than belonging to the AlkB-like NAOX subfamily of 2OG oxygenases, TYW5 belongs to the JmjC oxygenase subfamily (31,32), most of which act as histone *N*^ε^-methyl lysine demethylases and lysyl or arginyl hydroxylases. Most of the JmjC oxygenases, including TYW5, use a lysine (derived from DSBH βIV) and a tyrosine (derived from a β-strand involved in extending the major β-sheet positioned anti-parallel to DSBH βI) for 2OG C5-carboxylate binding (Figure 4g). In contrast to the differences observed in 2OG C5-carboxylate binding between the NAOX and JmjC enzymes, they all use a conserved binding mechanism for 2OG C1-carboxylate binding. An asparagine, present in the active site of ALKBH5 (Asn193), interacts with the C1-carboxylate oxygen of 2OG (Figure 4a–d and f) and is conserved not only in other NAOXs, but also in many other 2OG oxygenases including some JmjC domain oxygenases [i.e. JMJD2A (78)].

The active site of ALKBH5 appears more open than that of FTO. This may be due to a combination of the longer NRL in FTO as well as its C-terminal domain, both of which act to enclose the active site (Figure 3b). Whether this apparent difference is reflective of differences in m^6^A RNA substrates accepted by either FTO or ALKBH5 is unknown. It may be that ALKBH5 accepts bulkier RNA secondary structure in its active site, although preliminary results with a stem loop-derived RNA substrate suggest otherwise (12).

Both m^6^A demethylases, ALKBH5 and FTO, have a basic residue (Lys132 and Arg96, respectively) (Figure 4a and c) in the active site, which might be involved in substrate recognition/selection and/or product release following demethylation. Interestingly, it was shown that Lys132 can be acetylated from proteomics studies using deacetylase inhibitors in MV4-11 cells, a human acute myeloid leukaemia cell line (79); acetylation of Lys132 likely affects activity. The Arg96Met and Arg96Trp FTO variants almost completely abolish FTO enzymatic activity (39), supporting a crucial role for the conserved basic residue at this position in the m^6^A demethylases.

Arg283 is adjacent to the Fe(II) binding site in ALKBH5. An arginine residue at this position is conserved in all other structurally characterized NAOXs (27,28,36,39,40) (Figure 4a–f), and in many other 2OG oxygenases including deacetoxycephalosporin-C synthase and the related oxidase isopenicillin N synthase (80,81). This conserved arginine is proposed to be involved in oxygen activation (14,16), and its substitution has been shown to abrogate activity in the case of ALKBH3 (28). Furthermore, there is an acidic residue usually positioned near the active site in NAOXs (Glu175 in ALKBH2, Glu195 in ALKBH3, Glu234 in FTO and Asp135 in AlkB), which is present in ALKBH5 as Glu153 (Figure 4a–e). In the case of FTO, Glu234 has been shown to be important in substrate recognition. The Glu234Pro variant in FTO abolishes enzymatic activity (39). The AlkB Asp135Ala variant abolishes activity towards m^1^A in ssDNA, however, this variant increases activity towards m^1^G (82).

### Substrate recognition/selection elements

All structurally characterized NAOXs (AlkB, ALKBH2, ALKBH3, ALKBH8 and FTO) possess a conserved NRL (Figures 1a and 3a–f). The observed NRL (residues 124–161) of ALKBH5 comprises mixed β and loop secondary structure forming two β-hairpin-like loops: NRL1 (residues 124–137, strands β2–3) and NRL2 (residues 138–161, strands β4–5). In ALKBH5, NRL1 extends the major β-sheet of the DSBH and forms a short type I β turn (residues 127–130). NRL2 is partially disordered at the apex (residues 145–149) and is sandwiched between DSBH strand βII and the C-terminus (Figure 3a). Interestingly, the majority of the NRL sequence is observed to be disordered in the ALKBH8 crystal structure (Figure 3f), potentially indicating a requirement for flexibility in substrate binding (40). NRL1 is shorter in ALKBH5 than for the other NAOXs (AlkB, ALKBH2, ALKBH3 and FTO) (Figure 3a). The conserved ALKBH5 residue Arg130 in NRL1 (Figure 1d) may interact with the substrate phosphate backbone as was observed for the equivalent residue in the ALKBH2:dsDNA complex (Arg110); the ALKBH5 Arg130 equivalent Arg131Ala variant in ALKBH3 was shown to be inactive (28). The sequence of the disordered apex of ALKBH5 NRL2 contains two basic residues, Lys147 and Arg148, both of which are conserved across ALKBH5 homologues in various organisms (Figure 1d) and may be important for substrate recognition through interactions with the RNA substrate phosphate backbone.

ALKBH5, AlkB, FTO and ALKBH2 share a YXY/F motif positioned on the first strand of NRL2 (β4 in ALKBH5, ALKBH2 and FTO and on the equivalent of the ALKBH5 ‘anti-parallel’ strand β5 in AlkB) (Figure 1d). In the ALKBH2–dsDNA complex structure (PDB ID 3BUC), the hydroxyl of the proximal Tyr122 interacts with the *N*^6^ atom of the m^1^A substrate (3.2 Å) (Figure 4b) (27). Although the artificial covalent substrate attachment used to obtain the ALKBH2–DNA complex structure might slightly alter its position in the active site, the observed distance suggests a potential role for Tyr122 in catalysis. In ALKBH2 and FTO, the distal Phe124 or Tyr108 π-stacks directly with the base, and substitution of Tyr108 in FTO abolishes the activity (39). This ‘base stacking’ role is partially replaced by Trp69 in AlkB, aided by the proximal Tyr76 (taking the place of the distal Tyr141 in ALKBH5 due to its position on the anti-parallel strand β5) (Figure 4f), which makes interactions with Trp69 and the base as well as the phosphate backbone. The position of the NRL2 varies in the different NAOXs, possibly conferring specificity towards modified-base type and/or sequence context.

**Figure 5.**
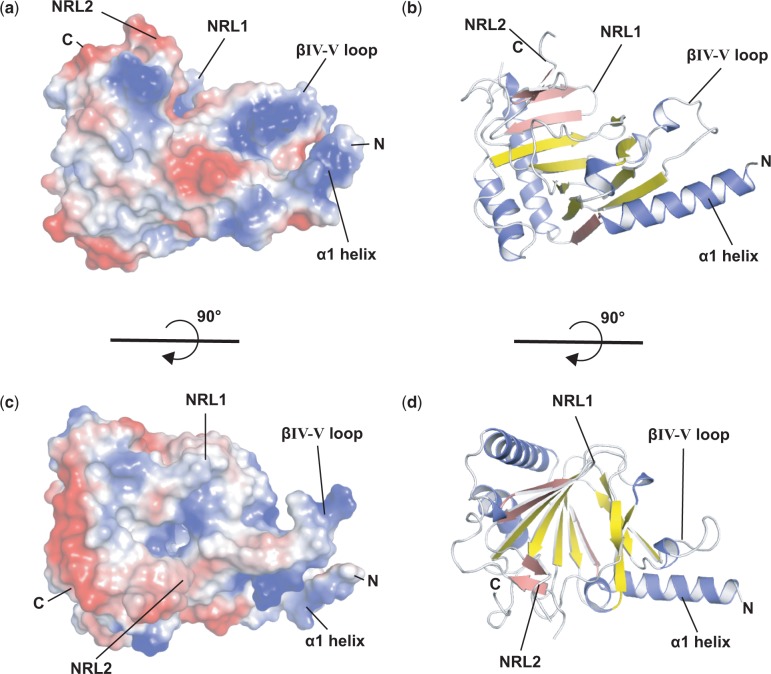
(**a** and **c**) ALKBH5 electrostatic surface representation (basic in blue; acidic in red) with 90° rotation along the X-axis and (**b** and **d**) corresponding ribbon representation. The substrate-binding groove around the active site is largely basic for the binding of the negatively charged ssRNA phosphate backbone. The basic region between the βIV-V loop and α1-helix forms a potential substrate binding groove.

The βIV–V loop of ALKBH5 contains basic residues (Lys231, Lys235 and Arg238) and overlaps with the complementary ‘unrepaired’ strand for dsDNA when superimposed with ALKBH2–dsDNA structure, potentially conferring ssRNA selectivity (Figure 3h). In ALKBH5, the βIV–V loop includes a 3_10_-helix that is absent in the other ALKBHs. The 3_10_-helix contains a solvent-exposed phenylalanine, Phe234, which might act as a phenylalanine finger to flip the m^6^A base into the active site by inserting between flanking bases (see below). The βIV–V loop, along with the α1-helix, appears to form a positively charged groove, which could be important for binding the negatively charged ssRNA phosphate backbone (Figure 5a–d). A similar ssRNA selectivity role has been proposed for a loop in FTO, the L1 loop, which is located between DSBH strands I and II (39). When ALKBH5 and FTO are superimposed, the L1 loop of FTO is situated in the same relative position near the active site as the βIV–V loop of ALKBH5 (Figure 3h). AlkB also has a βIV–V loop, although not as long as that observed in ALKBH5. In AlkB, Arg161 is located at the apex of the βIV–V loop. The AlkB Arg161Ala variant shows a decrease in affinity for methylated DNA, but its rate of activity was not affected. Thus, AlkB Arg161 is believed to play a role in the recognition of damaged bases (82).

### Model of substrate binding

Based on the structural information available for NAOX substrate complexes, we manually docked and energy-minimized two different modes of substrate binding to ALKBH5. Because ALKBH5 prefers ssRNA as a substrate, we modelled the consensus sequence 5′-GGm^6^ACU-3′ obtained by m^6^A-seq (8,9). The 5′–3′ direction of the strand through the active site was kept, consistent with that observed for the DNA in complex with AlkB and ALKBH2 (27,51). Two base-flipping modes, one with the flanking bases directly stacking against each other (as observed for ssDNA bound to AlkB) (51) and one with the insertion of a phenylalanine finger, Phe234, between the flanking bases (as observed for ALKBH2) (27). We restrained the docking to preserve the predicted interaction between Arg130 and the phosphate backbone (as observed in the ALKBH2–dsDNA complex) (27) and set the m^6^A substrate *N*^6^-methylgroup-metal distance to ∼4.1 Å, which is the average ‘substrate carbon to enzyme metal distance’ observed for most 2OG oxygenase–substrate structures (13). Tyr141 was placed in a position to interact with the phosphate backbone as for the AlkB–dsDNA complex (51). After manually adjusting for the two known base-flipping mechanisms, the models were energy-minimized to correct for geometry and steric interactions. The preliminary results suggest that the phenylalanine finger base-flipping mode involving Phe234 from the βIV–V loop intercalating the bases flanking m^6^A (Supplementary Figure S9) is more likely than the direct base-stacking mode. Although Phe234 appears relatively distant from the substrate binding groove, its position could change on substrate binding in an induced-fit mechanism aided by reduction of the Cys230–Cys267 disulfide (Supplementary Figure S4).

## CONCLUSIONS

The discovery that RNA *N*-methylation is reversible has opened up new avenues both for developing an understanding of the regulation of gene expression and for its medicinal exploitation. To date, the only oxygenases reported to remove m^6^A in RNA are ALKBH5 and FTO. Both enzymes appear to be physiologically important: FTO is associated with obesity, whereas ALKBH5 is involved in fertility (12,43). Although ALKBH5 reveals conserved structural elements with FTO, including a similar general active site chemistry and the use of an NRL, there are also clear differences. Work with other 2OG oxygenases (52) suggests these differences can be exploited in the development of selective compounds that can be used to test the validity of ALKBH5 and FTO as medicinal chemistry targets.

## ACCESSION NUMBERS

PDB ID 4NJ4.

## SUPPLEMENTARY DATA

Supplementary Data are available at NAR Online.

## FUNDING

The Biotechnology and Biological Sciences Research Council (BBSRC), the Wellcome Trust, the European Union, Pfizer Ltd. and The Rhodes Trust (J.S.S.). Funding for open access charge: The Biotechnology and Biological Sciences Research Council (BBSRC).

*Conflict of interest statement*. None declared.

## Supplementary Material

Supplementary Data
