# Supplementary material for: Structure of human RNA N6-methyladenine demethylase ALKBH5 provides insights into its mechanisms of nucleic acid recognition and demethylation
Source: Nucleic Acids Res. 2014 Jan 30;42(7):4741–54. doi: 10.1093/nar/gku085 (PMC3985658; doi:10.1093/nar/gku085)
Supplement: Supplementary Data [file supp_42_7_4741__index.html]

Structure of human RNA N6-methyladenine demethylase ALKBH5 provides insights into its mechanisms of nucleic acid recognition and demethylation — Structure of human RNA N6-methyladenine demethylase ALKBH5 provides insights into its mechanisms of nucleic acid recognition and demethylation — Supplementary Data 

# Structure of human RNA *N*6-methyladenine demethylase ALKBH5 provides insights into its mechanisms of nucleic acid recognition and demethylation

## Supplementary Data

files

**Files in this Data Supplement:**

- Supplementary Data - pdf file
