# Supplementary material for: Structure of human RNA N6-methyladenine demethylase ALKBH5 provides insights into its mechanisms of nucleic acid recognition and demethylation
Source: Nucleic Acids Res. 2014 Jan 30;42(7):4741–54. doi: 10.1093/nar/gku085 (PMC3985658; doi:10.1093/nar/gku085)
Supplement: Supplementary Data [file supp_gku085_nar-03570-h-2013-File008.pdf]

Structure of human RNA N^6-methyladenine demethylase ALKBH5 provides insights into its mechanisms of nucleic acid recognition and demethylation

WeiShen Aik[1], John S. Scotti[1], Hwanho Choi[1], Lingzhi Gong[1], Marina Demetriades[1], Christopher J. Schofield[1], and Michael A. McDonough[1,*]

[1] Chemistry Research Laboratory, University of Oxford, 12 Mansfield Road, Oxford, OX1 3TA, United Kingdom
* To whom correspondence should be addressed. Tel: +44 01865 275 629; Fax: +44 01865 285 002; Email: michael.mcdonough@chem.ox.ac.uk

SUPPLEMENTARY INFORMATION

Table of Contents

1. Activity of ALKBH5$_{66\text{-}292}$
2. Inhibition of ALKBH5$_{66\text{-}292}$
3. Crystal packing
4. Surface cysteines and a disulphide on ALKBH5 structure
5. MS/MS spectrum of ALKBH5$_{66\text{-}292}$ sample from crystallisation drop
6. Superimposition of FTO-IOX3 active site on ALKBH5 active site
7. Relative position of IOX3 with respect to the 2OG binding pocket in ALKBH5
8. Proposed IOX3 – Cys200 reaction mechanism
9. 5-mer ssRNA substrate modelling

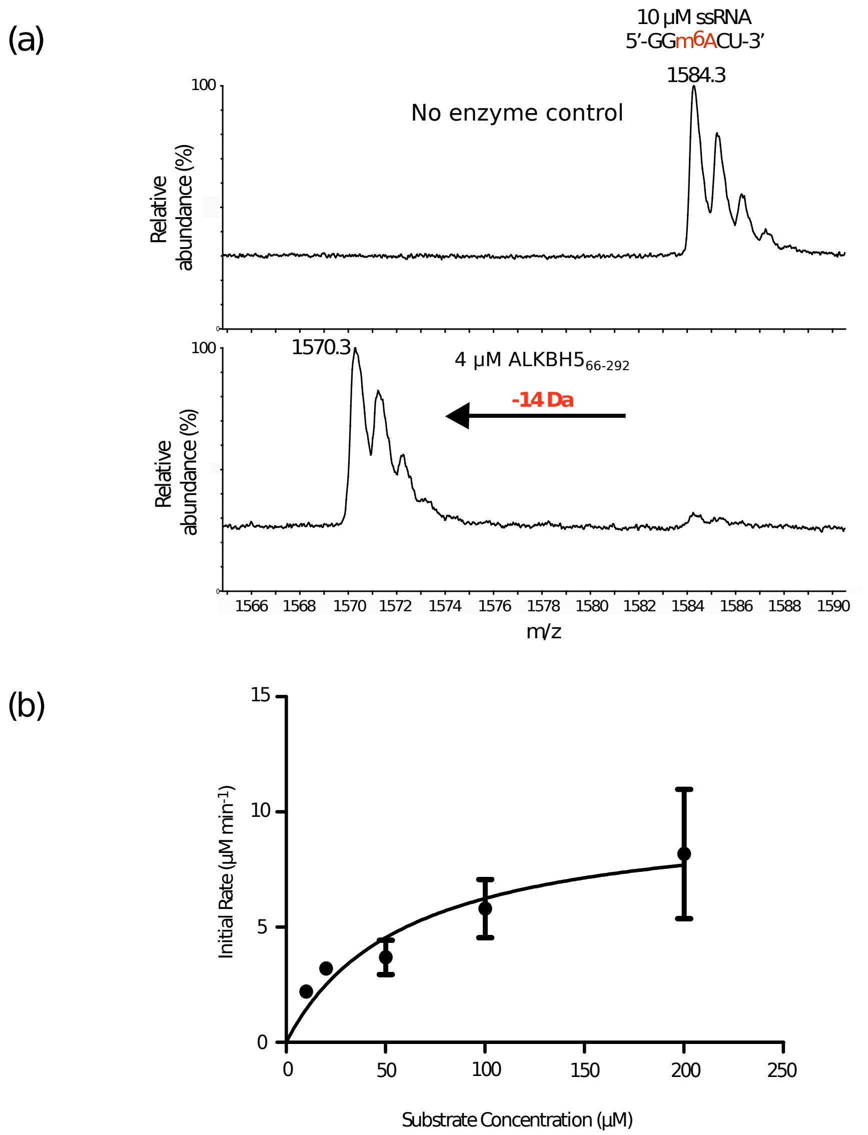

Figure S1. Activity assays of ALKBH5$_{66\text{-}292}$. (a) MALDI spectra showing demethylation (-14 Da) of 10 µM 5-mer ssRNA (5'-GGm^{6}ACU-3') (1584 Da) in the presence of 4 µM ALKBH5$_{66\text{-}292}$ after a 20 min reaction. (b) Michaelis-Menten analyses of initial rate (µM·min^{-1}) using a 5-mer ssRNA substrate (µM) and 4 µM ALKBH5$_{66\text{-}292}$ give substrate K_m of 60 ± 28 µM and V_{max} of 10 ± 1.8 µM·min^{-1}.

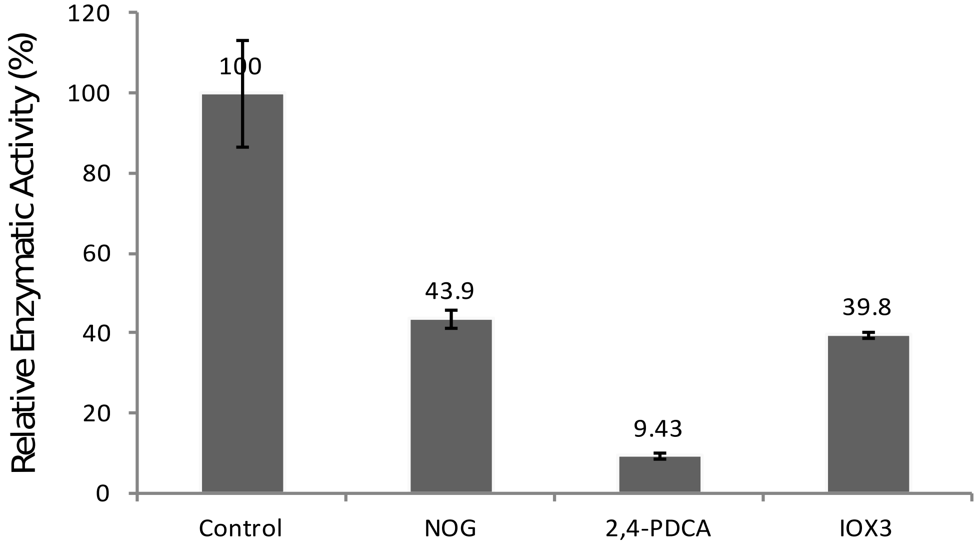

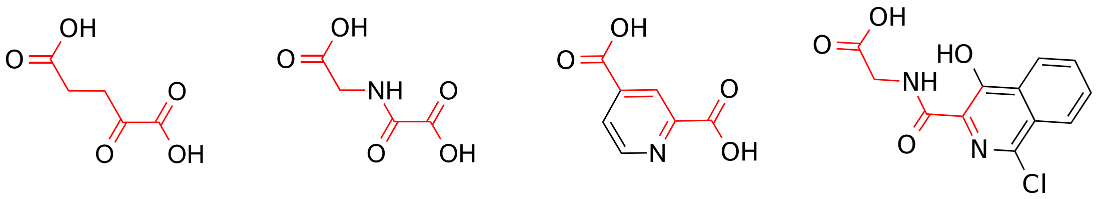

2-oxoglutarate (2OG)

***N*-oxalylglycine (NOG)**

2,4-pyridine dicarboxylate (2,4-PDCA)

(1-chloro-4-hydroxyisoquinoline-3-carbonyl)glycine (IOX3)

Figure S2. Inhibition assays of ALKBH5$_{66\text{-}292}$. Percentage residual activity of ALKBH5$_{66\text{-}292}$ relative to no inhibitor control after a 5 min reaction in the presence of 150 μM *N*-oxalylglycine (NOG), 150 μM 2,4-pyridine dicarboxylic acid (2,4-PDCA), and 150 μM IOX3. Demethylation assays were conducted in triplicate and errors are standard deviations of the mean (n=3). Chemical structures of cosubstrate 2OG and the mimetic inhibitors used are shown. Portions of inhibitors isosteric to cosubstrate 2OG are highlighted as red bonds.

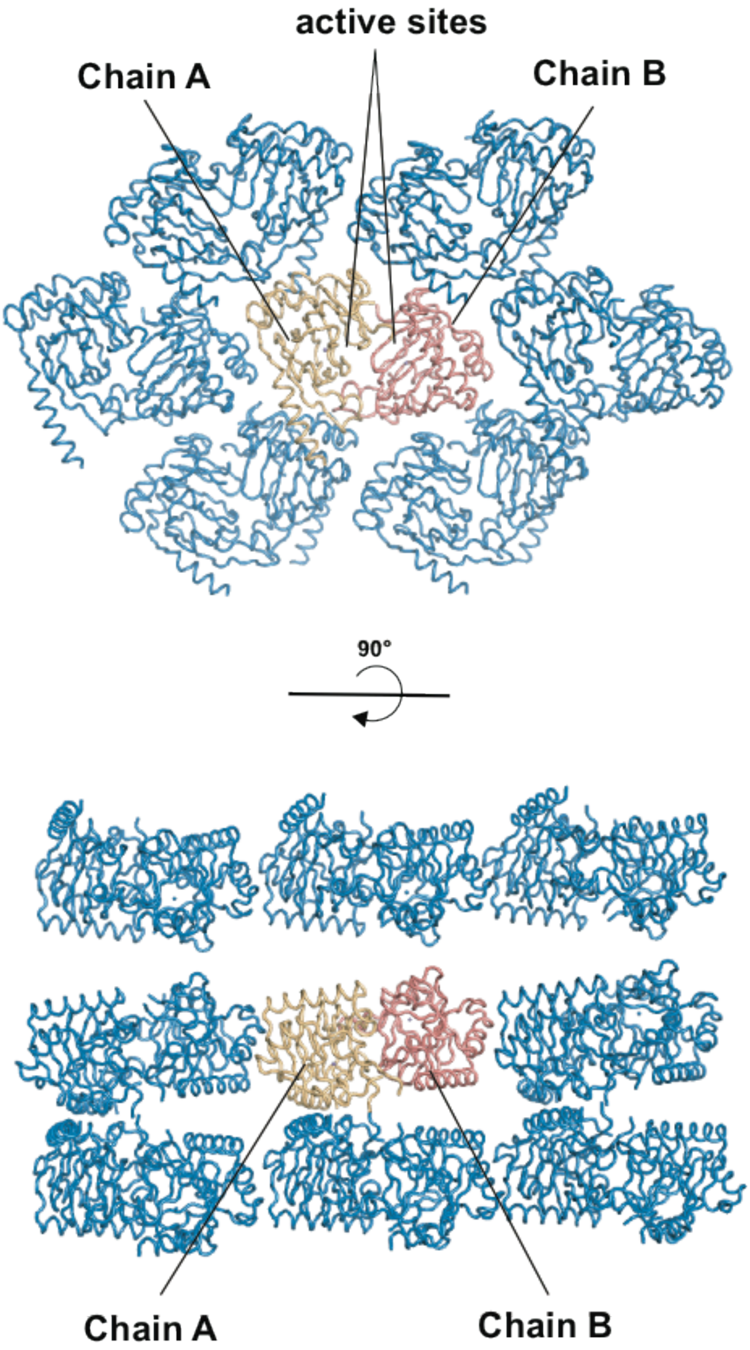

Figure S3. Crystal packing of ALKBH5$_{66\text{-}292}$ protein molecules in two orientations rotated at 90° relative to each other. There are two ALKBH5 molecules in the asymmetric unit.

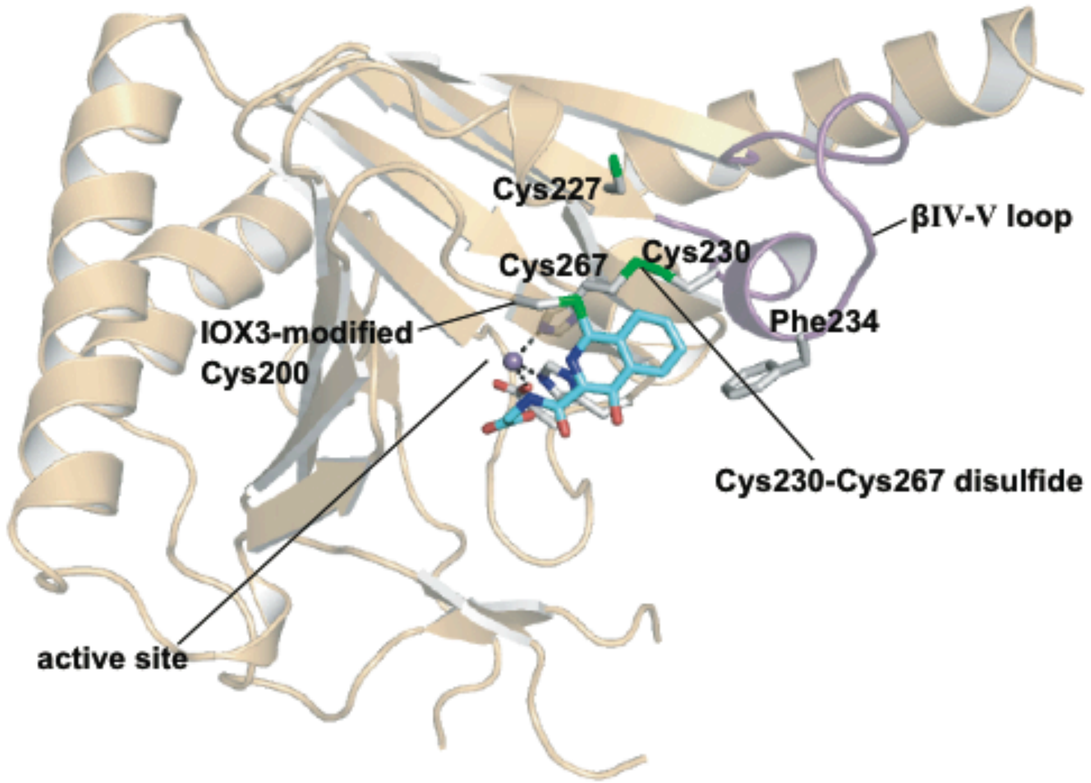

Figure S4. Surface cysteine-residues of ALKBH5. Cys230 and Cys267 form a disulphide bond. Cys200 is modified by IOX3. Reduction of the Cys230-Cys267 disulphide bond might result in a more flexible βIV-V loop for induced-fit substrate binding.

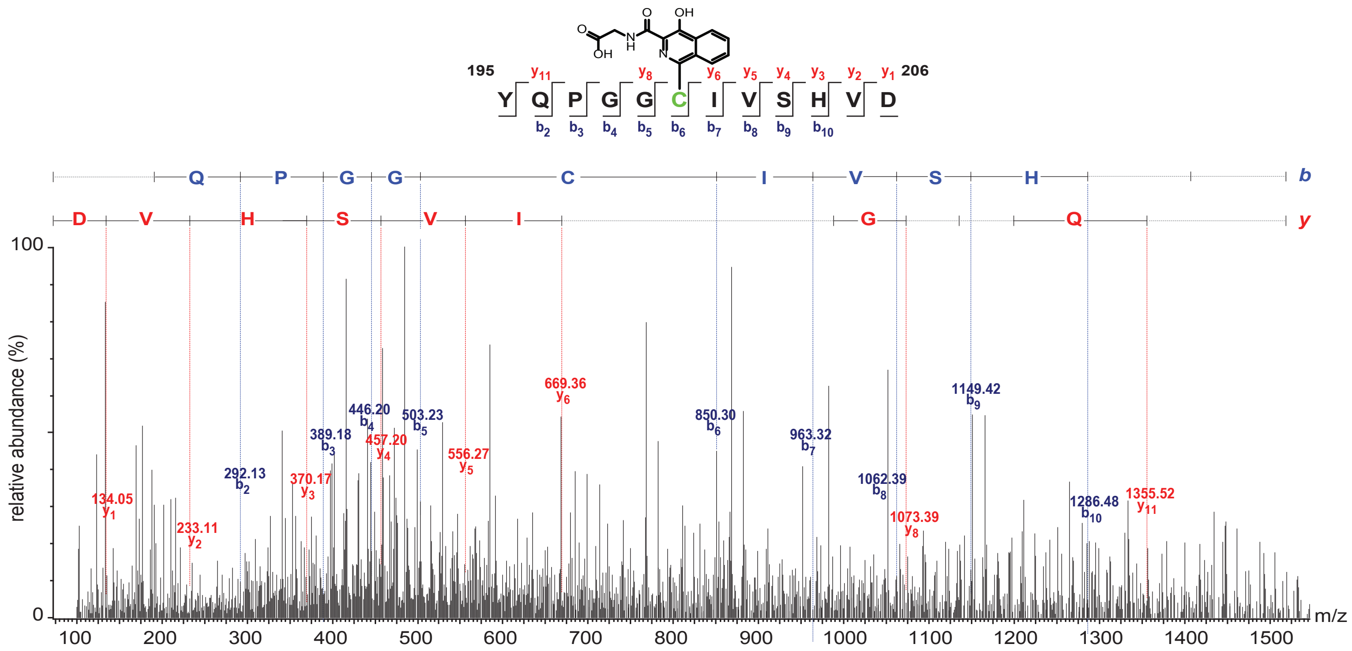

Figure S5. MS/MS spectrum of ALKBH5 crystal from a crystallisation drop that was incubated for 16 weeks. A peak at m/z=850.30 corresponding to a b ion of YQPGGC-IOX3 is observed.

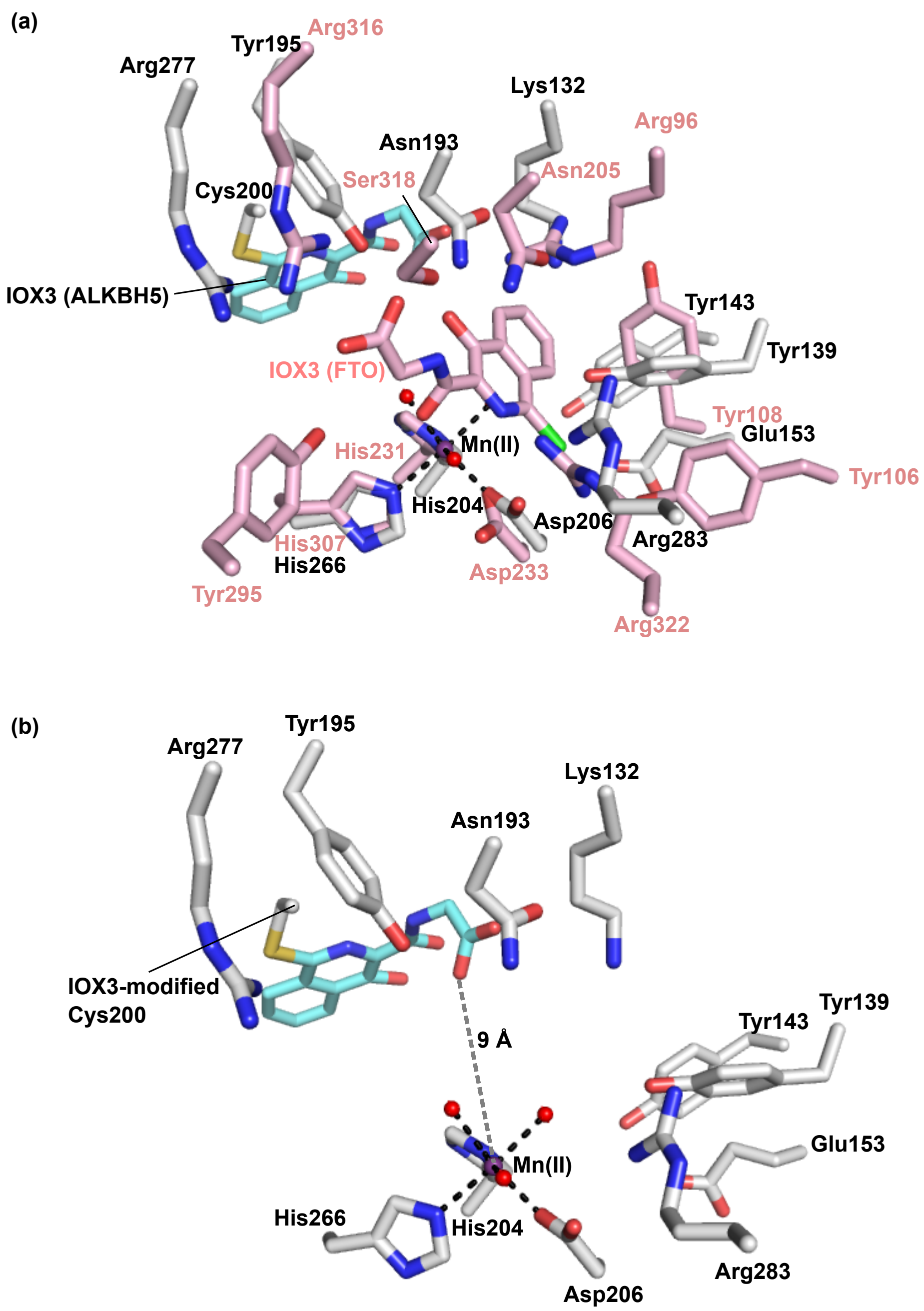

Figure S6. Superimposition of the FTO-IOX3 complex structure (residues and IOX3 coloured pink) (PDB ID 4IE6) with the ALKBH5 structure (residues coloured white, IOX3 coloured cyan). (b) Active site residues of ALKBH5 showing the position of the IOX3-modified Cys200. Note: in the crystallographically observed mode of IOX3, it is not possible for it to coordinate to the active site metal as observed for FTO (41).

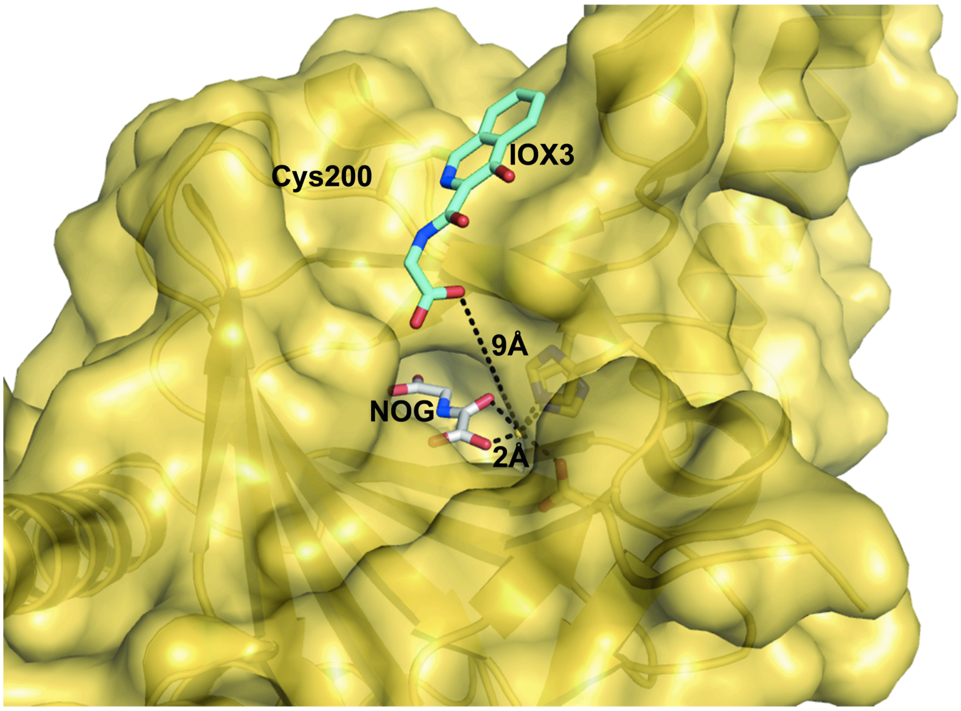

Figure S7. Overall structure of ALKBH5 showing the relative position of IOX3 with respect to the 2OG binding pocket. NOG (grey sticks) is modelled into the 2OG binding pocket based on the FTO-NOG structure (4IDZ) (41).

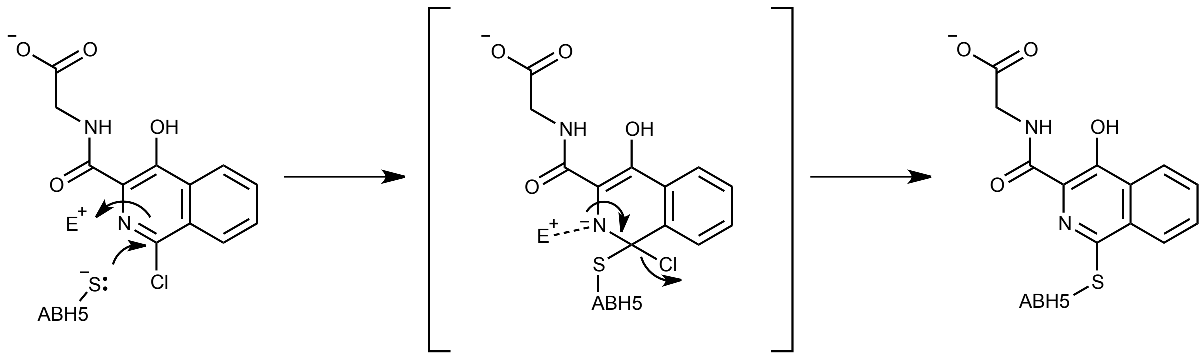

Figure S8. Proposed aromatic nucleophilic substitution mechanism for the crystallographically observed covalent modification of Cys200 by IOX3.

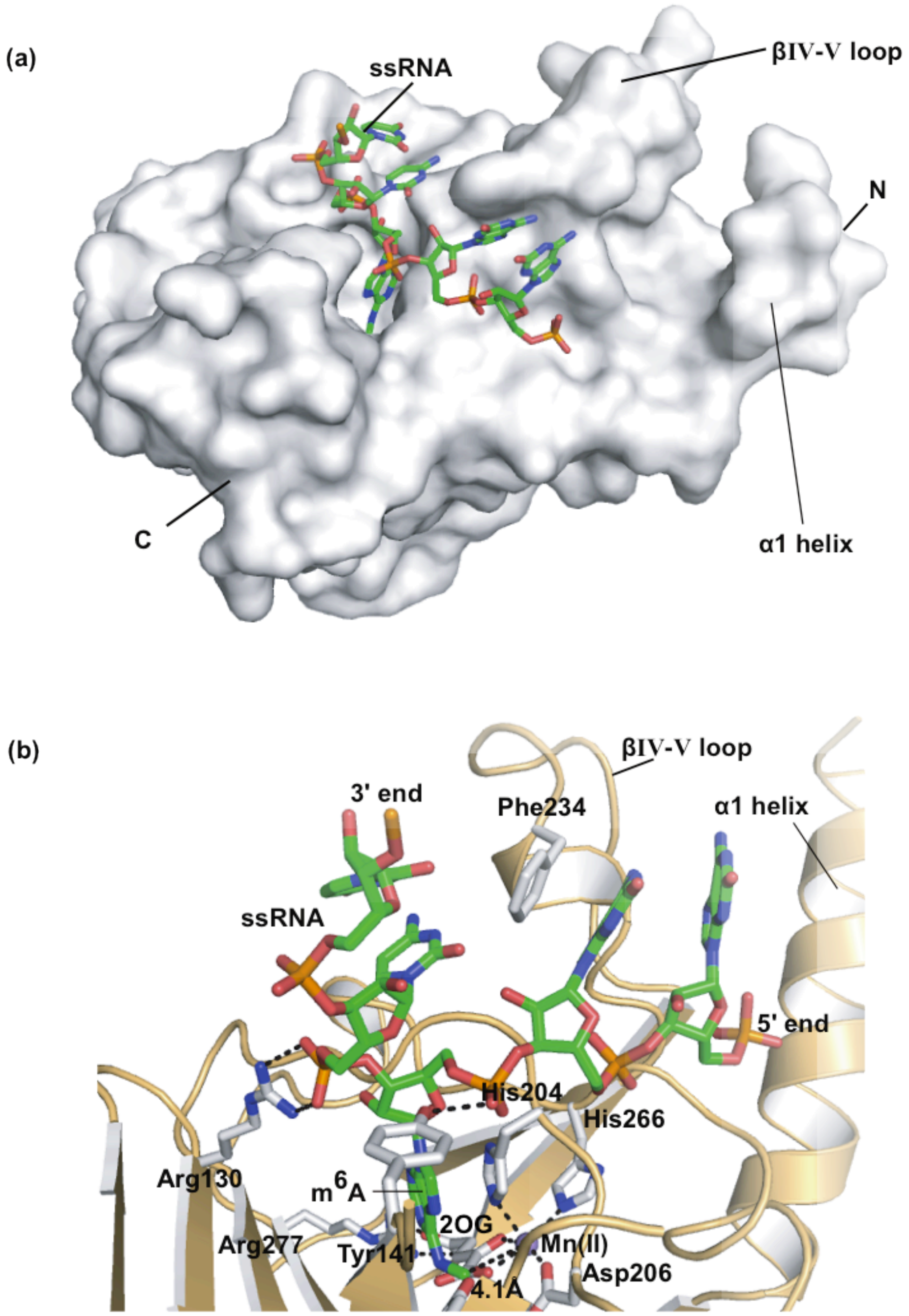

Figure S9. Model of substrate binding to the active site of ALKBH5. (a) surface representation and (b) cartoon representation with modelled 5-mer ssRNA 5'-GGm^{6}ACU-3'. The structure of ALKBH2-dsDNA (PDB ID 3BUC) and its phenylalanine base-flipping mode were used as a template for ALKBH5 substrate modelling. The substrate was manually docked onto the ALKBH5 structure followed by energy minimisation.
